# RETRACTED: Ma et al. MiR-122 Induces Radiosensitization in Non-Small Cell Lung Cancer Cell Line. *Int. J. Mol. Sci.* 2015, *16*, 22137–22150

**DOI:** 10.3390/ijms26041614

**Published:** 2025-02-14

**Authors:** Debin Ma, Hui Jia, Mengmeng Qin, Wenjie Dai, Tao Wang, Erguang Liang, Guofu Dong, Zuojun Wang, Zhiyuan Zhang, Fan Feng

**Affiliations:** 1 Department of Respiratory Diseases, General Hospital of Shenyang Military Command, Shenyang 110016, China; 2 Department of Pharmacy, General Hospital of Shenyang Military Command, Shenyang 110016, China; 3 Department of Pharmacy, Beijing Chuiyangliu Hospital Affiliated to Tsinghua University, Beijing 100022, China; 4 Institute of Toxicology and Pharmacology, Medicine Military Medical Science Academy of the Chinese PLA, Beijing 100850, China; 5 Institute of Radiation, Medicine Military Medical Science Academy of the Chinese PLA, Beijing 100850, China

The Journal retracts the article “MiR-122 Induces Radiosensitization in Non-Small Cell Lung Cancer Cell Line” [1].

Following publication, concerns were brought to the attention of the Editorial Office regarding a potential duplication of figures in this paper [1].

Adhering to our procedure, an investigation was conducted by the Editorial Office and the Editorial Board that confirmed the duplication of two panels within Figure 2a listing different experimental time points.

While the authors fully cooperated with the Editorial Office during the investigation, they were unable to satisfactorily explain the overlap of these figures, nor could they meet the required quality standards of raw images in order to consider a correction as per the journal original image requirements policy (https://www.mdpi.com/journal/ijms/instructions#oriimages). As a result, the Editorial Board and Editor-in-Chief were unable to confirm the reliability of the findings and subsequently decided to retract this paper this publication [1], as per MDPI’s retraction policy (https://www.mdpi.com/ethics#_bookmark30).

This retraction was approved by the Editor-in-Chief of the journal *Int. J. Mol. Sci.*

The authors did not provide a comment on this decision.
